# Improving Polar-Coded SCMA System by Information Coupling and Parity Check

**DOI:** 10.3390/s20236740

**Published:** 2020-11-25

**Authors:** Xi Wu, Yafeng Wang

**Affiliations:** Key Laboratory of Universal Wireless Communications, Ministry of Education, Beijing University of Post and Telecommunications, Beijing 100876, China; ericwx2729@163.com

**Keywords:** sparse code multiple access, polar code, partially information coupling, parity check, successive cancellation list, extrinsic information transfer

## Abstract

In this paper, the uplink information-coupled polar-coded sparse code multiple access (PC-SCMA) system is proposed. For this system, we first design the encoding method of systematic joint parity check and CRC-aided (PCCA) polar code. Using the systematic PCCA-polar code as base code, the partially information-coupled (PIC) polar code is constructed. Then, a joint iterative detection and successive cancellation list (SCL)-decoding receiver is proposed for the PC-SCMA system. For the receiver, the coupled polar decoder’s extrinsic messages are calculated by the Bayes rule and soft cancellation (SCAN) algorithm. Based on the extrinsic information transfer (EXIT) idea, the PIC PCCA-polar code is optimized. Simulation results demonstrate that the PIC PCCA-PC-SCMA system outperforms the other polar (or LDPC) coded SCMA systems at various code rates and channel configurations. Additionally, compared with an uncoupled PC-SCMA system with SCL decoder, the complexity of PIC PCCA-PC-SCMA is reduced at a high $E_{b}/N_{0}$

## 1. Introduction

With the rapid increasing demand of the future mobile wireless network, new requirements are put forward for the communication technologies and the applications. The typical application scenarios of the fifth generation (5G) wireless networks consist of enhanced Mobile BroadBand (eMBB), massive Machine Type Communications (mMTC) and Ultra-Reliable and Low Latency Communications (URLLC). For 5G scenarios, the non-orthogonal multiple access (NOMA) has been proposed to increase the system throughput and accommodate massive communication connectivity. Moreover, as opposed to orthogonal multiple access (OMA), the NOMA has low transmission latency and signaling cost. Recently, many NOMA schemes were proposed for 5G, such as the sparse code multiple access (SCMA) [1], pattern division multiple access (PDMA) [2], multi-user shared access (MUSA) [3], low-density signatures (LDS) multiple access [4], and so on. Among the available NOMA schemes, SCMA is a coding-based multiplexing scheme, which can achieve an obvious increase in spectral efficiency compared to OMA. Its codes are mapped to multi-dimensional sparse codebooks for transmission. Due to the sparsity of the codebook, the message passing algorithm (MPA) [5] with moderate complexity can be used for multi-user detection (MUD).

Actually, the quality of service (QoS) of NOMA system can be realized by using effective channel coding. Turbo code and low-density parity-check (LDPC) code are studied in many existing NOMA systems for the error correction [6,7]. Furthermore, according to the results in [7], the new radio (NR) LDPC-coded NOMA schemes have almost the same block error rate (BLER) performance as the corresponding Turbo-coded NOMA schemes.

Polar code proposed by Arikan [8] is the first constructive code that provably achieves the symmetric capacity of binary-input memoryless channels (BMCs). In [8], Arikan proposed a hard-output successive cancellation (SC) decoder that achieved capacity in the limit of large block length; its performance with finite-length codes was less promising. Tal and Vardy proposed the successive cancellation list (SCL) algorithm whose performance is very close to that of a maximum likelihood decoder [9]. SCL decoding not only improves the block-error probability of polar code, but also enables one to use modified polar code constructed by concatenating a polar code with a cyclic redundancy check (CRC) code as an outer code [10,11]. The simulation results demonstrate that the short-block-size polar code with CRC-aided (CA) SCL has performance advantage over the corresponding LDPC code. Although all of these decoders offer better performance than the SC decoder, none provides the soft outputs essential for turbo-based receivers. Improving the performance of the SC decoder has been at the key of the related research while the generation of soft information. In [12], the belief propagation (BP) decoder is proposed to decode polar code. It has the advantages of having better performance than the SC decoder and providing soft outputs, but has very high storage and processing complexity. In [13], the authors developed a low-complexity soft-output version of the SC decoder called the soft cancellation (SCAN) decoder that produces reliability information for both the coded and message bits. The SCAN decoder achieves comparable performance with far less complexity and storage than the BP decoder. However, it cannot achieve the performance of SCL decoder with large list size. According to 3GPP TS 38.212 [14], polar code is elected as the standard coding technique for the control channel in support of the eMBB service, since it has an excellent error-correction capability based on SCL decoding, especially for short-length packet transmissions.

With the abundant research results of polar code, the application of polar code in NOMA system has also made progress. In [15], a polar-coded SCMA (PC-SCMA) was proposed, but there is no iteration between the MPA detector and the polar decoder. However, for practical implementations, the iterative detection between the multiuser detector and the channel decoder can improve the performance of the SCMA system. In [16], a three-stage channel transform structure of the polar-coded NOMA system was proposed, and a joint successive cancellation (JSC) detection and decoding scheme was designed. Although the JSC algorithm improves the performance of the system, it increases the processing latency. In [17], based on the joint factor graph of SCMA and polar code, the authors have proposed a joint iterative detection and decoding (JIDD) receiver based on SCAN algorithm. The JIDD without inner iterations has best performance when compared with the general iterative detection and decoding (IDD) scheme. It improves the performance of the polar-coded sparse code multiple access (PC-SCMA) system significantly while also saving the complexity costs. However, since the performance of SCAN decoder is not good enough, this JIDD receiver is not ideal. In particular, the performance of the PC-SCMA system is worse than that of LDPC-coded SCMA (LDPC-SCMA) at low code rate. In order to improve the performance of PC-SCMA, the design of the code’s structure and decoding algorithm are feasible.

One approach that leads to better performance is to exploit the benefits of long code through spatial coupling. Theoretically, an infinite long codeword chain can be constructed by coupling an infinite number of finite length code blocks (CBs) in a certain structure. For long data sequence transmission, a class of partially information-coupled (PIC) Turbo code [18] and PIC LDPC code [19] have been proposed. In these schemes, a transport block (TB) is segmented into several finite-length CBs under the practical decoding complexity and decoding latency constraints. Every two consecutive codes blocks in a TB are coupled by sharing a few information bits. Thus, PIC code can also be regarded as a class of spatially coupled code. PIC polar code was also constructed by systematic polar code in [20], and a windowed decoder based on CA-SCL algorithm is used for decoding. The TB error rate (TBER) results show that the PIC polar code achieves approximately 0.3 dB coding gain over the uncoupled counterparts for variable code rates. Importantly, the PIC polar code also outperforms the PIC Turbo code at a high $E_{b}/N_{0}$. Basis of this, in this paper, we consider improving the SCMA system by PIC polar code.

Though the code construction of PIC polar code is straightforward, it is very challenging to obtain the promised coding gain over SCMA system compared to [17]. First, the coding gain of PIC polar code proposed in [20] is limited. To achieve more coding gain, one straightforward approach is to improve the code block error rate (CBER) of each CB. In the 3GPP meetings, various “assistant bits + basic polar” schemes have been proposed. The idea of “using assistant bits for polar decoding” has been widely adopted, including CA-polar code [21], joint parity-check and CRC-aided (PCCA) polar code [22,23], Hash-CRC polar code [24], and distributed CRC-aided (DCA)polar code [25,26,27]. The various solutions differ in terms of number of assistant bits, their usage (for error detection and/or correction), positions, and values. Especially, the PCCA-polar code uses the parity check bits for error correction in SCL decoder and gets better performance than other codes. Simultaneously, the Hash-CRC polar code and DCA-polar code have comparable performance to the CA-Polar code. By the way, the systematic polar code that was used in [20] is CA-polar code. To improve the performance of PIC polar code, PCCA-polar code can be considered as the base code. However, the design of the PCCA-polar code in the systematic form must be resolved. Second, besides the feed-back decoding mechanism based on coupling information bits, the reason why the PIC polar code can obtain excellent performance is that it uses the SCL algorithm for the basic decoder. In iterative receiver, in order to iteratively exchange their messages between the constituent MPA detector and polar decoder, they must be soft-input-soft-output (SISO). Therefore, the hard-output decoding algorithms such as SCL cannot be directly applied to the iterative receiver. The calculation of the extrinsic messages of the coupled polar decoder remains to be resolved. Third, with the coupled polar decoder, increasing the basic decoder’s list size can improve the performance of the system, but it also increases the decoding complexity. On the premise of guaranteeing the performance, the complexity of the receiver should be reduced as far as possible.

On the basis of above discussing, the main contributions of this paper can be summarized as follows:

- The uplink information-coupled PC-SCMA system is proposed. At the transmitter, channel coding, SCMA mapping, and transmission are all performed based on CB. When the entire TB is received, the receiver performs iterative detection and decoding.
- The PCCA-polar code in system form is designed. Then, every two consecutive systematic PCCA-polar code blocks are connected by information coupling technology to form a new type of PIC polar code. For windowed decoder, the list algorithm based on parity check can provide more accurate extrinsic messages for the coupling CBs. In addition, the windowed decoding algorithm is improved for the requirement of iterative system.
- An extrinsic messages construction algorithm of coupled polar decoder is proposed. This algorithm enables the coupled polar decoder to exchange extrinsic messages with the MPA detector, and achieving iterative detection and decoding.
- A joint iterative detection and SCL decoding algorithm (JIDS) based on variable list size is designed. During the iteration, those CBs who failed CRC verification improved the performance in the next iteration by increasing the list size, while others maintained a smaller list size. In addition, when all CBs are correctly decoded, the iteration is stopped immediately.
- The extrinsic information transfer (EXIT) idea is used to optimize the weight factor of extrinsic messages construction algorithm and coupling ratio of PIC PCCA-polar code.

The rest of this article is organized as follows. In Section 2, the system model of uplink information-coupled PC-SCMA is described. In Section 3, the characteristics of systematic PCCA-polar code are analyzed, and the corresponding encoding algorithm is introduced. Then, the construction method of PIC PCCA-polar code is proposed. In Section 4, a joint iterative detection and SCL decoding algorithm with variable list size is proposed to reduce the complexity. The extrinsic messages of coupled polar decoder are constructed and applied to the receiver. Based on EXIT idea, the transfer characteristics of various polar decoders are analyzed, and the PIC PCCA-polar code is optimized in Section 5. Section 6 shows the simulation results and the complexity comparison. Finally, conclusions are reached in Section 7. To read this article better, we summarize the abbreviations in Table 1.

## 2. System Model 

**Figure 1:**
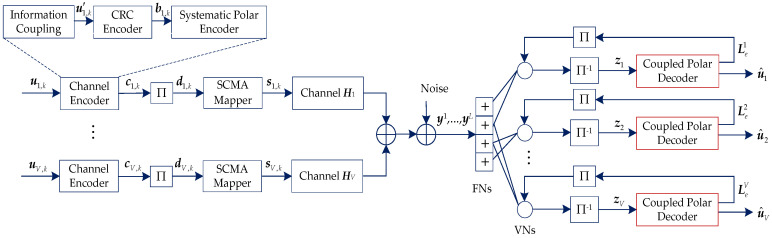
Uplink information-coupled polar-coded sparse code multiple access (PC-SCMA) system.

Figure 1 shows the uplink information-coupled PC-SCMA system. In this system, a TB consists of $L_{c}$ mutually coupled CB. For CB *k*, $1\leq k\leq L_{c}$, the information bits of *V* users $U_{k}=\left\{u_{1,k},u_{2,k},\cdots ,u_{V,k}\right\}$ are encoded to $C_{k}=\left\{c_{1,k},c_{2,k},\cdots ,c_{V,k}\right\}$ by information coupling, CRC, and systematic encoder. For user *v*, its information bits and coded bits are denoted as $u_{v,k}=\left\{u_{v,k}^{1},u_{v,k}^{2},\cdots ,u_{v,k}^{K}\right\}$ and $c_{v,k}=\left\{c_{v,k}^{1},c_{v,k}^{2},\cdots ,c_{v,k}^{N}\right\}$, respectively. Then, each user’s codeword is interleaved by the random interleaver, which is denoted by $d_{v,k}=\Pi \left(c_{v,k}\right)$.

**Figure 2:**
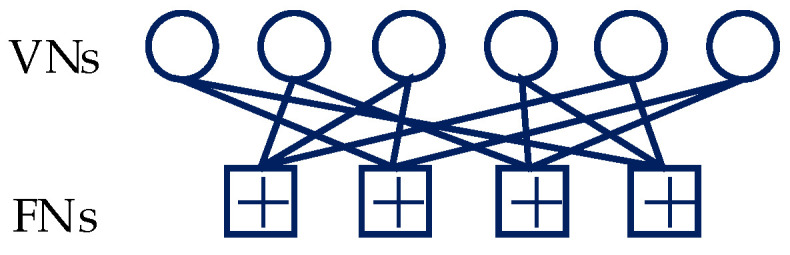
SCMA factor graph with six users and four resources.

Assuming the *V* users multiplexed over *M* (*M* < *V*) orthogonal resources. Then, every $Q=\log_{2}\left(M\right)$ bits of $d_{v,k}$ are mapped to a *J*-dimensional complex codeword $s_{v,k}=\left\{s_{v,k}^{1},s_{v,k}^{2},\cdots ,s_{v,k}^{J}\right\}$ by SCMA mapper, and the number of SCMA codewords is $L_{k}=N/Q$. Thus, the overloading factor of SCMA is defined as λ=V/J and the structure of an SCMA code can be represented by a J×V binary mapping matrix ***F***. As shown in Figure 2, a SCMA factor graph with six variable nodes (VNs) and four function nodes (FNs) is illustrated, where VNs and FNs represent the users and resources, respectively. The corresponding mapping matrix is denoted as

(1) $$F=\left[\begin{array}{cccccc} 0 & 1 & 1 & 0 & 1 & 0\\ 1 & 0 & 1 & 0 & 0 & 1\\ 0 & 1 & 0 & 1 & 0 & 1\\ 1 & 0 & 0 & 1 & 1 & 0 \end{array}\right].$$

The iterative detection and decoding are performed after the whole TB is obtained by the receiver. Thus, the number of SCMA codeword $s_{v}=\left\{s_{v,1},\ldots ,s_{v,L_{c}}\right\}=\left\{s_{v}^{1},\ldots ,s_{v}^{L}\right\}$ is $L=L_{c}N/Q$. Supposing the channel gains matrix between the base station and user *v* at the *l*-th block is denoted as

(2) $$H_{v}^{l}=\left[\begin{array}{cccc} h_{v}^{l,1} & & & \\ & h_{v}^{l,2} & & \\ & & \ddots & \\ & & & h_{v}^{l,J} \end{array}\right],$$

where $h_{v}^{l,j}$ represents the channel gain between user *v* and resource *j* at the *l*-th block, and $H_{v}^{l}$ is a diagonal matrix. Thus, at the receiver, the *l*-th received symbols can be described as

(3) $$y_{l}=\sum_{v=1}^{V}H_{v}^{l}s_{v}^{l}+n_{l},$$

where 1≤l≤L, $y_{l}={\left\{y_{l,1},y_{l,2},\cdots ,y_{l,J}\right\}}^{T}$, and $s_{v}^{l}={\left\{s_{v}^{l,1},s_{v}^{l,2},\cdots ,s_{v}^{l,J}\right\}}^{T}$. $n_{l}={\left\{n_{l,1},n_{l,2},\cdots ,n_{l,J}\right\}}^{T}$ is a complex additive white Gaussian noise (AWGN) vector with the mean vector 0 and covariance matrix $N_{0}I$, and $N_{0}$ denotes the variance of Gaussian noise. 

At the receiver, the received symbols $y_{l}$ are decoded by the MPA detector first. The output extrinsic messages of the MPA detector are in the form of log-likelihood ratio (LLR), and de-interleaved to $z_{v}=\left\{z_{v,1},\ldots ,z_{v,L_{c}}\right\}$ for user *v*, where $z_{v,k}=\left\{z_{v,k}^{1},\ldots ,z_{v,k}^{N}\right\}$. Then, as the a priori information, $z_{v}$ is feed into the coupled polar decoder. The output extrinsic messages $L_{e}^{v}$ is then interleaved and entered into the MPA detector as a priori information. After several iterations, the receiver converges and outputs each user’s decoded information bits $\left\{{\hat{u}}_{1},{\hat{u}}_{2},\cdots ,{\hat{u}}_{V}\right\}$, where ${\hat{u}}_{v}=\left\{{\hat{u}}_{v,1},\ldots ,{\hat{u}}_{v,L_{c}}\right\}$. The information-coupled polar coding is described in Section 3. In addition, the CRC is used not only for SCL decoding, but also for constructing extrinsic messages and iterative reception. The detailed algorithm is given in Section 4.

## 3. Design of Partially Information-Coupled Polar Code based on Parity Check

PIC codes are constructed by sharing a few systematic information bits between every two consecutive codes blocks. Following [20], the PIC polar code constructed by systematic CA-polar code achieved considerable coding gains over the uncoupled code. In this section, we focus on further improving the performance of the PIC polar code. 

### 3.1. Systematic PCCA-Polar Code

According to 3GPP TS 38.212, based on the idea of “using parity check bits for polar decoding,” PCCA-polar code has been adopted for the uplink control channel coding since it prevents premature deletion of the correct candidate path in list decoding. For short-length packet, PCCA-polar code exhibits superior performance compared to CA-polar code. The performance of PIC polar code should be further enhanced by using PCCA-polar code as the base code. However, PCCA-polar code, in its standard form, is a non-systematic code. In other words, the information bits do not appear as part of the codeword transparently. Therefore, the traditional PCCA-polar code cannot be directly used to construct the PIC polar code. 

Since any linear code can be turned into a systematic code, [28] gives encoding and decoding methods for the systematic polar code that preserves the same frame error rate. However, this method does not work for all types of polar codes, such as PCCA-polar code and DCA-polar code. In this subsection, we give the encoding method of systematic PCCA-polar code as follows.

**Figure 3:**
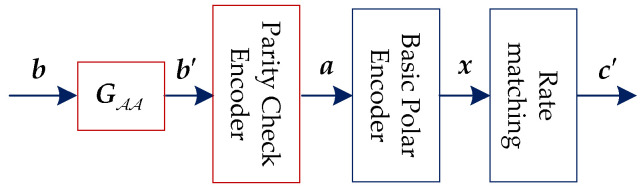
Systematic parity check and CRC-aided (PCCA)-polar encoder.

For systematic PCCA-polar code, the encoding is performed as Figure 3. The information block (including CRC bits) is defined as $b=\left\{b_{1},b_{2},\ldots ,b_{K^{\prime }}\right\}$, and A denotes the information indices, where $\left|A\right|=K^{\prime }$. As presented in [8], the recursive construction of the generator matrix can be defined as $G={\left[\begin{array}{cc} 1 & 0\\ 1 & 1 \end{array}\right]}^{⊗\log_{2}\left(N_{m}\right)}$, where $N_{m}$ is the mother code length and ⊗ denotes the Kronecker product. $G_{AA}$ is the submatrix of G consisting of the array of elements $\left(G_{i,j}\right)$ with i,j∈A, and similarly for the other submatrices. The module with parameter $G_{AA}$ can implement the mapping $b\mapsto b^{\prime }$ by computing $b^{\prime }=bG_{AA}$. Then, by inserting parity check bits p and frozen bits 0 into $b^{\prime }$, $a=\left\{a_{1},\ldots ,a_{N_{m}}\right\}$ can be obtained. The indices of parity check bits are denoted by $P_{c}$ and $\left|P_{c}\right|=n_{pc}$. Thus, the indices of information with assistant bits (including CRC bits and parity check bits) can be represent by $A^{\prime }=A\cup P_{c}$, where $A^{\prime }\subset \left\{1,\ldots ,N_{m}\right\}$ corresponds to the $K^{\prime }+n_{pc}$ most reliable indices (skipping shortened or punctured positions) and $a_{A^{\prime }}=\left\{b^{\prime },p\right\}$. Then, the basic polar encoding is performed by x=aG. Since $G_{AA}$ and $G_{A^{\prime }A^{\prime }}$ are lower-triangular with ones on the diagonal, they are invertible (in fact, the inverse of $G_{AA}$ and $G_{A^{\prime }A^{\prime }}$ are themselves). Thus, $x_{A}=b^{\prime }G_{AA}=b$ and $x_{A^{\prime }}=a_{A^{\prime }}G_{A^{\prime }A^{\prime }}$ can be get. It can be seen, the mother code x is systematic. Finally, $c^{\prime }$ is obtained by rate matching. Since $A^{\prime }$ is chosen by skipping shortened or punctured positions, $c^{\prime }$ is also systematic code. According to the preceding description, to ensure that $x_{A}=b$, the choice of $P_{c}$ is the key issue.

First, we give two theorems as follow.

**Theorem** **1.** *Denote* $g_{i}$ *as the i-th row of* $G_{A^{\prime }A^{\prime }}$ *and $1^{i}$. as the indices of ones in* $g_{i}$ *. If* $j\in 1^{i}$ *, there is $1^{j}\subseteq 1^{i}$.*

**Proof.**  Appendix A.  □

**Theorem** **2.** *Denote* ${A^{\prime }}_{i}$ *as the i-th element of* $A^{\prime }$ *,* $g_{i}$ *as the i-th row of* $G_{A^{\prime }A^{\prime }}$ *and* $1^{i}$ *as the indices of ones in* $g_{i}$ *. Assume* ${A^{\prime }}_{i}\in P_{c}$ *and* $j\in 1^{i}$ *, if and only if* ${A^{\prime }}_{j}\in P_{c}$ *, polar code is systematic.*

**Proof.**  According to Theorem 1, if $j\in 1^{i}$, the relation $1^{j}\subseteq 1^{i}$ holds. Obviously, there is $1^{j}\cap 1^{i}=1^{j}$. If ${A^{\prime }}_{j}\notin P_{c}$, ${A^{\prime }}_{j}$ is an information index. By the transformation $x_{A^{\prime }}=a_{A^{\prime }}G_{A^{\prime }A^{\prime }}$, the information bits $\left\{x_{A^{\prime }}^{k},k\in 1^{j}\right\}$ must be superimposed with parity bit $a_{A^{\prime }}^{i}$. Thus, the systematic code could not be obtained.  □

**Figure 4:**
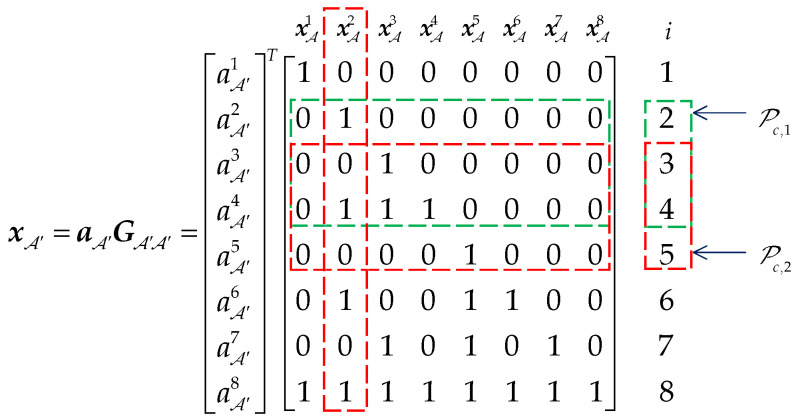
The effect of $P_{c}$ on transformation $x_{A^{\prime }}=a_{A^{\prime }}G_{A^{\prime }A^{\prime }}$.

For example, let $N_{m}=16$, $A^{\prime }=\left\{8,10,11,12,13,14,15,16\right\}$, and the corresponding submatrix $G_{A^{\prime }A^{\prime }}$ is shown in Figure 4. If ${A^{\prime }}_{i}={A^{\prime }}_{4}$ is chosen as an element of $P_{c}$, the corresponding position set of ones can be expressed as $1^{4}=\left\{2,3,4\right\}$. According to Theorem 1, the relationship $1^{2}=\left\{2\right\}\subset 1^{4}$ and $1^{3}=\left\{3\right\}\subset 1^{4}$ can be deduced. Following Theorem 2, ${A^{\prime }}_{2}$ and ${A^{\prime }}_{3}$ must be the elements of $P_{c}$. Thus, when $P_{c,1}=\left\{{A^{\prime }}_{2},{A^{\prime }}_{3},{A^{\prime }}_{4}\right\}$ is chosen as the parity check indices, the sequence $a_{A^{\prime }}=\left\{{b^{\prime }}_{1},p_{1},p_{2},p_{3},{b^{\prime }}_{2},{b^{\prime }}_{3},{b^{\prime }}_{4},{b^{\prime }}_{5}\right\}$. From Figure 4, the parity check bits $p=\left\{a_{A^{\prime }}^{2},a_{A^{\prime }}^{3},a_{A^{\prime }}^{4}\right\}$ do not affect the information bits $\left\{x_{A^{\prime }}^{1},x_{A^{\prime }}^{5},x_{A^{\prime }}^{6},x_{A^{\prime }}^{7},x_{A^{\prime }}^{8}\right\}$ by transformation $x_{A^{\prime }}=a_{A^{\prime }}G_{A^{\prime }A^{\prime }}$, in other words, $x_{A}=\left\{x_{A^{\prime }}^{1},x_{A^{\prime }}^{5},x_{A^{\prime }}^{6},x_{A^{\prime }}^{7},x_{A^{\prime }}^{8}\right\}=\left\{b_{1},b_{2},b_{3},b_{4},b_{5}\right\}$ and x is systematic code. However, for $P_{c,2}=\left\{{A^{\prime }}_{3},{A^{\prime }}_{4},{A^{\prime }}_{5}\right\}$ and $a_{A^{\prime }}=\left\{{b^{\prime }}_{1},{b^{\prime }}_{2},p_{1},p_{2},p_{3},{b^{\prime }}_{3},{b^{\prime }}_{4},{b^{\prime }}_{5}\right\}$, the information bit $x_{A^{\prime }}^{2}$ is superimposed with parity bit $p_{2}=a_{A^{\prime }}^{4}$, so $x_{A}=\left\{x_{A^{\prime }}^{1},x_{A^{\prime }}^{2},x_{A^{\prime }}^{6},x_{A^{\prime }}^{7},x_{A^{\prime }}^{8}\right\}=\left\{b_{1},b_{2}⊕p_{2},b_{3},b_{4},b_{5}\right\}$ and x is not the systematic code.

According to Theorems 1 and 2 and 3GPP TS 38.212, given $A^{\prime }$ and $n_{pc}$, Algorithm 1 is proposed to choose $P_{c}$.
**Algorithm 1** Get parity check indices $P_{c}$**Input**: $A^{\prime }$ and $n_{pc}$.**Output**: $P_{c}$.  1: Initialize: $P_{c}=Ø$.  2: Generate submatrix $G_{A^{\prime }A^{\prime }}$ and calculate the row’s weight $W_{t}=\left\{W_{t}^{1},\ldots ,W_{t}^{\left|A^{\prime }\right|}\right\}$ of $G_{A^{\prime }A^{\prime }}$.  3: $w_{t}=1$.  4: **while**
$\left|P_{c}\right|<n_{pc}$
**do**  5: S=Ø.  6: **for**
$i=1\to \left|A^{\prime }\right|$
**do**
  7: **if**
$W_{t}^{i}=w_{t}$
**then**  8: Get $1^{i}$ of $g_{i}$.  9: $S^{\prime }=\left|1^{i}\cap S\right|$.10: **if**
$\left|1^{i}\right|-\left|S^{\prime }\right|=1$
**then**11: update the set $S=S\cup \left\{{A^{\prime }}_{i}\right\}$.12: **end if**13: **end if**14: **end for**15: **if**
$n_{pc}-\left|P_{c}\cup S\right|<0$
**then**16: Select $n_{pc}-\left|P_{c}\right|$ elements with the lowest reliability of S as set $S^{\prime \prime }$.17: update the set $P_{c}=P_{c}\cup S^{\prime \prime }$.18: **else**19: update the set $P_{c}=P_{c}\cup S$.20: **end if**21: $w_{t}=w_{t}+1$.22: **end while**

Finally, we can give the encoding method of systematic PCCA-polar code in Algorithm 2. The rate-matching pattern ℰ is generated according to 3GPP TS 38.212.
**Algorithm 2** Systematic PCCA-polar encoding**Input**: b, $N_{m}$, $A^{\prime }$, $n_{pc}$ and rate-matching pattern ℰ, where $\left|ℰ\right|=N_{e}$.**Output**: $c^{\prime }$.  1: Initialize: Set a *p*-length cyclic shift register y[0],…,y[p−1] to 0, and a=0, where $\left|a\right|=N_{m}$.  2: Generate the generator matrix G.  3: Get the information and CRC indices $A=\left\{A^{\prime }\backslash P_{c}\right\}$, and generate the submatrix $G_{AA}$.  4: Calculate $b^{\prime }=bG_{AA}$.  5: **for**
$i=1\to N_{m}$**do** //PC bits generation [14]  6: Cyclic left shift the register.  7: If i∈A: set $y\left[0\right]=a_{i}⊕y\left[0\right]$.  8: If $i\in P_{c}$: set $a_{i}=y\left[0\right]$.  9: **end for**10: Perform the basic polar coding to obtain mother code by x=aG.11: Get systematic PCCA-polar codes $c^{\prime }$ by rate-matching pattern ℰ.

### 3.2. Partially Information-Coupled PCCA-Polar Code

In [20], the authors have constructed a PIC polar code based on systematic CA-polar code. Multiple systematic polar codes blocks are concatenated to form a large code frame by sharing a few information bits between every two consecutive CBs. More specifically, dummy bits are inserted into the first CB and the last CB to terminate the frame. To improve the PIC polar code, in this subsection, we introduce a new type of PIC polar code based on systematic PCCA-polar code. However, following the encoding method presented in [20], the dummy bits are punctured in the end, and the length of the last CB is different from other CBs. More importantly, the windowed decoding of TB will be terminated when a CB is error decoded. Thus, the un-decoded CBs could not supply the extrinsic message for iterative system, such as SCMA. Therefore, we propose a new construction and decoding method for our PIC PCCA-polar code as follows.

**Figure 5:**
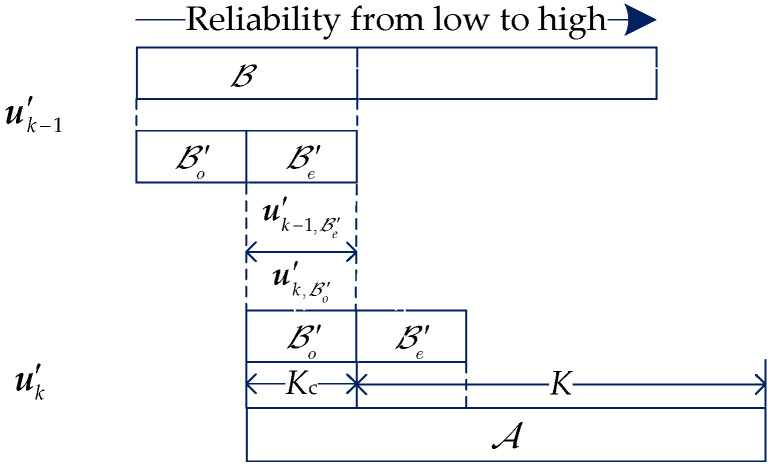
The coupling relation between every two consecutive code blocks (CBs).

Let $u=\left\{u_{1},u_{2},\ldots ,u_{L_{c}}\right\}$ denote the information sequence of a TB, where $L_{c}$ is the number of CBs, $u_{k}=\left\{u_{k}^{1},u_{k}^{2},\ldots ,u_{k}^{K}\right\}$ corresponding to the *k*-th CB. Let A denote the information indices with length $K+K_{c}$ for the coupled information block ${u^{\prime }}_{k}$. $B=\left\{B_{o},B_{e}\right\}$ expresses the coupling indices, where $B_{o}$ and $B_{e}$ correspond to the subset with odd and even indices of B, respectively. The indices of $B_{o}$ and $B_{e}$ in A are respectively denoted as ${B^{\prime }}_{o}$ and ${B^{\prime }}_{e}$. Figure 5 shows the coupling relation between every two consecutive CBs.

**Figure 6:**
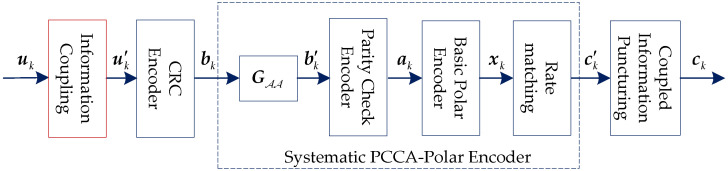
Partially information-coupled (PIC) PCCA-polar encoder.

For the first CB (*k* = 1), an all-zero dummy block with length $K_{c}$ is inserted into indices ${B^{\prime }}_{o}$ of ${u^{\prime }}_{1}$, and get ${u^{\prime }}_{1}=\left\{0,u_{1}\right\}$. For $1<k\leq L_{c}$, the information bits that are on the indices ${B^{\prime }}_{e}$ of the (*k*−1)-th CB are inserted into the position ${B^{\prime }}_{o}$ of the *k*-th CB to obtain ${u^{\prime }}_{k}=\left\{{u^{\prime }}_{k-1,B^{\prime }{}_{e}},u_{k}\right\}$, where ${u^{\prime }}_{k,B^{\prime }{}_{o}}={u^{\prime }}_{k-1,B^{\prime }{}_{e}}$. Thus, the CBs have been coupled by sharing a few information bits between every two consecutive CBs. Then, for every CB, the coupled information block ${u^{\prime }}_{k}$ is attached to the CRC bits and encoded by systematic PCCA-polar encoder as shown in Figure 6. For $1\leq k\leq L_{c}$, the sequence $x_{k}$ satisfies $x_{1,B_{o}}=0$ and $x_{k,B_{o}}=x_{k-1,B_{e}}$. Finally, $x_{k,B_{o}}$ is punctured to obtain $c_{k}$.

In this scheme, dummy block is not transmitted, and coupled information blocks are only transmitted once even though each of them is shared by two consecutive CBs. Hence, the effective code rate of the PIC PCCA-polar code is R=K/N, where *N* is the code length of CB. In addition, we define $\delta =K_{c}/K$ as coupling ratio of the proposed PIC PCCA-polar code, and the mother code rate is $r=\left(K+K_{c}\right)/N_{m}$.

In [20], the authors proposed a windowed decoding based on the look-back and go-back scheme for PIC polar code. The algorithm is divided into feed-forward decoding and feed-back decoding. When the *k*-th CB is decoded correctly in the feed-forward decoding, the (*k*−1)-th CB can be corrected by the perfect extrinsic messages of the *k*-th CB in feed-back decoding. This decoding scheme is also suitable for our PIC PCCA-polar code. However, since the iterative system requires complete extrinsic messages, subsequent CBs’ decoding cannot be terminated even if there is a CB decoding error for our decoding scheme. The CBs with feed-forward decoding failure were corrected only once by feed-back decoding.

## 4. Joint Iterative Detection and SCL Decoding Receiver

In this section, the extrinsic message of the coupled polar decoder is constructed and the details of JIDS algorithm are presented.

In [17], the authors proposed a JIDD receiver for the uplink PC-SCMA system. The MPA detector outputs its extrinsic messages within one inner-loop iteration and directly as the input prior messages of SCAN decoder, and vice versa. The receiver only needs outer iterations and no inner iterations of constituent SCMA detector and SCAN decoder are needed. Since the performance of SCAN decoder is not ideal, the performance of the PC-SCMA system is limited. Therefore, this subsection proposes a JIDS algorithm for the PIC PC-SCMA system.

To better describe the algorithm, we define some notations as follows:

- $M_{g_{i}\to q_{j}}$: The messages passing from the *i*-th function node $g_{i}$ to the *j*-th variable node $q_{j}$.
- $M_{q_{j}\to g_{i}}$: The messages passing from the *j*-th variable node $q_{j}$ to the *i*-th function node $g_{i}$.
- $Q_{j}$: The set of function nodes that connect to variable node $q_{j}$.
- $G_{i}$: The set of variable nodes that connect to function node $g_{i}$.
- $\left\{Q_{j}\backslash i\right\}$: Excluding function node $g_{i}$ from the set of $Q_{j}$.
- $\left\{G_{i}\backslash j\right\}$: Excluding variable node $q_{j}$ from the set of $G_{i}$.
- $P\left(s_{v}^{l}\right)$: The prior information of the *l*-th SCMA codeword for user v.

### 4.1. Update Function Nodes of MPA Detector

As described in Section 2, the iterative detection and decoding are performed after the whole TB’s signal $\left\{y_{1},\ldots ,y_{l},\ldots ,y_{L}\right\}$ is obtained by the receiver. The function node $g_{i}$ of the MPA detector updates its information and pass it to its neighboring variable nodes. The messages passing from the *i*-th function node $g_{i}$ to the *j*-th variable node $q_{j}$ can be calculated by

(4) $$M_{g_{i}\to q_{j}}\left(s_{v}^{l}\right)=\sum_{s_{t}^{l},t\in \left\{G_{i}\backslash j\right\}}\left\{P\left(y_{l,j}|s_{v}^{l},s_{t}^{l},t\in \left\{G_{i}\backslash j\right\}\right)\cdot \prod_{t\in \left\{G_{i}\backslash j\right\}}M_{q_{t}\to g_{i}}\left(s_{t}^{l}\right)\right\}.$$

Then, the bits extrinsic message of the MPA detector can be expressed as

(5) $$P_{e,\mathrm{MPA}}^{v,\left(l-1\right)Q+m}\left(x\right)=\sum_{s_{v,x}^{l}\in \left\{s_{v}^{l}|q_{v}^{m}=x\right\}}\prod_{t\in Q_{j}}M_{g_{t}\to q_{j}}\left(s_{v,x}^{l}\right),1\leq m\leq Q.$$

where $\left\{s_{v}^{l}|q_{v}^{m}=x\right\}$ is the SCMA codewords set whose elements satisfy the mapping relationship $\left\{g_{v}:\left(q_{v}^{1},\cdots ,q_{v}^{m-1},x,q_{v}^{m+1},\cdots ,q_{v}^{Q}\right)\to s_{v}^{l}\right\}$.

Thus, in the form of LLR, the extrinsic bit messages of the MPA detector can be expressed as

(6) $$L_{e,\mathrm{MPA}}^{v,\left(l-1\right)Q+m}\left(x\right)=\ln\frac{P_{e,\mathrm{MPA}}^{v,\left(l-1\right)Q+m}\left(x=0\right)}{P_{e,\mathrm{MPA}}^{v,\left(l-1\right)Q+m}\left(x=1\right)}.$$

As the prior information of the coupled polar decoder, the extrinsic messages of the MPA detector $L_{e,\mathrm{MPA}}^{v}=\left\{L_{e,\mathrm{MPA}}^{v,1},\ldots ,L_{e,\mathrm{MPA}}^{v,k},\ldots ,L_{e,\mathrm{MPA}}^{v,L_{c}}\right\}$ is de-interleaved to $z_{v}=\left\{z_{v,1}\ldots ,z_{v,k},\ldots ,z_{v,L_{c}}\right\}$, where $L_{e,\mathrm{MPA}}^{v,k}=\left\{L_{e,\mathrm{MPA}}^{v,1},\cdots ,L_{e,\mathrm{MPA}}^{v,N}\right\}$ and $z_{v,k}$ are expressed by

(7) $$z_{v,k}=\Pi^{-1}\left(L_{e,\mathrm{MPA}}^{v,k}\right),1\leq k\leq L_{c}.$$

### 4.2. Updated a Priori Information of MPA Detector

The prior information of MPA detector is updated by the extrinsic messages of coupled polar decoder. In this subsection, we mainly focus on the SCL decoding algorithm of base polar code. Note that in iterative receiver, in order to iteratively exchange their messages between the MPA detector and channel decoder, they should be soft-input-soft-output. Therefore, the coupled polar decoder must reconstruct the soft output information that can be used for iteration. On the basis of LLR-based SCL algorithm [11], we introduce the construction of extrinsic messages of the SCL decoder and extend it to the coupled polar decoder.

For the *k*-th CB of user *v*, after the decoding of the last bit, the SCL decoder outputs the $L_{o}$ most likely path ${\hat{a}}_{v,k}^{l^{\prime }}$ and corresponding path metric $P_{v,k}^{l^{\prime }}$, $1\leq l^{\prime }\leq L_{o}$. For each candidate path $l^{\prime }$, the corresponding codeword can be obtained by encoding the estimated sequence ${\hat{a}}_{v,k}^{l^{\prime }}$.

(8) $${\hat{c}}_{v,k}^{l^{\prime }}={\hat{a}}_{v,k}^{l^{\prime }}G.$$

In order to calculate the extrinsic messages, the path metric $P_{v,k}^{l^{\prime }}$ is normalized as follows

(9) $$p_{v,k}^{l^{\prime }}=\exp\left(P_{v,k}^{l^{\prime }}\right)/\sum_{l^{\prime }=1}^{L_{o}}\exp\left(P_{v,k}^{l^{\prime }}\right).$$

Then, the probability of the bit $c_{v,k}^{i}$ can be written as

(10) $$p\left(c_{v,k}^{i}\right)=\sum_{\begin{array}{l} 1\leq l^{\prime }\leq L_{o}\\ {\hat{c}}_{v,k}^{i,l^{\prime }}=c_{v,k}^{i} \end{array}}p_{v,k}^{l^{\prime }}.$$

Thus, for the mother code $x_{k}$, by the Bayes rule, the extrinsic messages of the SCL decoder $L_{x,k}^{v}=\left\{L_{x,k}^{v,1},\ldots ,L_{x,k}^{v,N_{m}}\right\}$ can be calculated as

(11) $$L_{x,k}^{v,i}\left(c_{v,k}^{i}\right)=\ln\left(\frac{p\left(c_{v,k}^{i}=0\right)}{p\left(c_{v,k}^{i}=1\right)}\right)=\{\begin{array}{cc} +\infty & p\left(c_{v,k}^{i}=1\right)=0\\ \ln\left(\frac{\sum_{\begin{array}{l} 1\leq l^{\prime }\leq L_{o}\\ {\hat{c}}_{v,k}^{i,l^{\prime }}=0 \end{array}}p_{v,k}^{l^{\prime }}}{\sum_{\begin{array}{l} 1\leq l^{\prime }\leq L_{o}\\ {\hat{c}}_{v,k}^{i,l^{\prime }}=1 \end{array}}p_{v,k}^{l^{\prime }}}\right) & p\left(c_{v,k}^{i}=1\right)\neq 0\&p\left(c_{v,k}^{i}=0\right)\neq 0\\ -\infty & p\left(c_{v,k}^{i}=0\right)=0 \end{array}.$$

With CRC aiding, if the path $l^{\prime }$ passes the CRC detection, the sign of $L_{x,k}^{v,i}$ is modified by

(12) $$L_{x,k}^{v,i}=\left(1-2{\hat{c}}_{v,k}^{i,l^{\prime }}\right)\left|L_{x,k}^{v,i}\right|.$$

We call the method described above a Bayes construction (BC) algorithm, which can be directly used in list decoder. But, the accuracy of the extrinsic message depends on the list size and the result of CRC check. When the list size is small and the check fails, the constructed extrinsic messages of SCL decoding by the BC algorithm are not accurate enough. Therefore, the hybrid construction (HC) algorithm can be used to construct the extrinsic messages of the SCL decoder. When the CRC check fails, the SCAN algorithm is used to construct the extrinsic message as follows.

**Figure 7:**
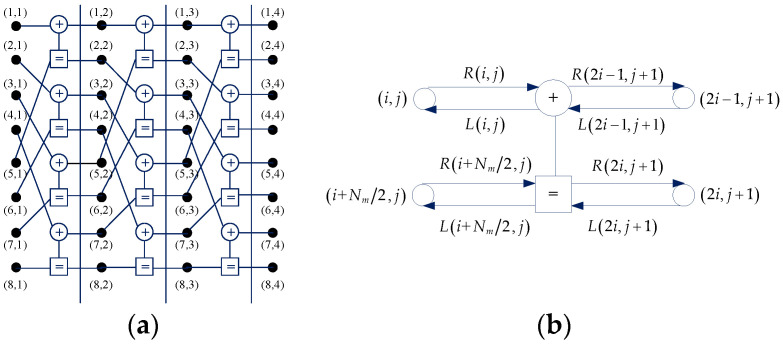
Factor graph and processing element. (**a**) Factor graph; (**b**) processing element.

Based on the SCAN algorithm, the extrinsic messages are calculated on the factor graph. For a $N_{m}$-length polar mother code, the factor graph is composed of $n+1=\log_{2}\left(N_{m}\right)+1$ columns, and every two adjacent columns comprise $N_{m}/2$ processing elements (PEs) shown in Figure 7. The pair (i,j) refers to the *i*-th variable node at column *j* in the factor graph, where $1\leq i\leq N_{m}$ and 1≤j≤n+1. The left and right propagating messages of node (i,j) are denoted as L(i,j) and R(i,j), respectively.

The left messages and right messages are passed toward the left and right direction of the factor graph, respectively. The L(i,n+1) and R(i,1) are respectively initialized by the prior LLR information $z_{v,k}$ and (13).

(13) $$R\left(i,1\right)=\{\begin{array}{ccc} 0 & & i\in A^{\prime }\\ +\infty & & i\in {A^{\prime }}^{c} \end{array}.$$

From Figure 7b, the messages of each node are updated on a PE by the following recursive formulas:

(14) $$L\left(i,j\right)=f\left(L\left(2i,j+1\right)+R\left(i+N_{m}/2,j\right),L\left(2i-1,j+1\right)\right),$$

(15) $$L\left(i+N_{m}/2,j\right)=L\left(2i,j+1\right)+f\left(R\left(i,j\right),L\left(2i-1,j+1\right)\right),$$

(16) $$R\left(2i-1,j+1\right)=f\left(L\left(2i,j+1\right)+R\left(i+N_{m}/2,j\right),R\left(i,j\right)\right),$$

(17) $$R\left(2i,j+1\right)=L\left(i+N_{m}/2,j\right)+f\left(R\left(i,j\right),L\left(2i-1,j+1\right)\right),$$

where f(⋅) is defined as

(18) $$f\left(a,b\right)=2\mathrm{arctanh}\left[\tanh\left(\frac{a}{2}\right)\cdot \tanh\left(\frac{b}{2}\right)\right].$$

**Figure 8:**
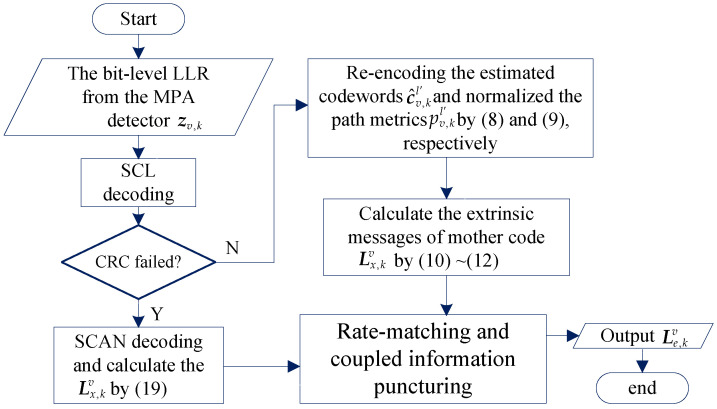
The flowchart of hybrid construction algorithm.

For the details of the SCAN algorithm, we refer to [13]. Then, following [17], after one iteration of SCAN decoding, the extrinsic messages of mother code can be expressed as

(19) $$L_{x,k}^{v,i}=R\left(i,n+1\right)+\alpha \frac{{\bar{R}}_{n+1}}{{\bar{L}}_{n+1}}L\left(i,n+1\right),$$

where ${\bar{R}}_{n+1}$ and ${\bar{L}}_{n+1}$ are the average values of R(i,n+1) and L(i,n+1), respectively. α is the weight factor. Finally, by the coding rules of PIC PCCA-polar code, the extrinsic messages of mother code $L_{x,k}^{v}$ is punctured or shortened to $L_{e,k}^{v}=\left\{L_{e,k}^{v,1},\ldots ,L_{e,k}^{v,N}\right\}$. The flowchart of hybrid construction algorithm is given by Figure 8.

Therefore, the extrinsic message of user *v* can be represent by $L_{e}^{v}=\left\{L_{e,1}^{v},\ldots ,L_{e,k}^{v},\ldots L_{e,L_{c}}^{v}\right\}$. Then, $L_{e}^{v}$ is interleaved to $L_{a}^{v}=\left\{L_{a,1}^{v},\ldots ,L_{a,k}^{v},\ldots ,L_{a,L_{c}}^{v}\right\}$ by $L_{a,k}^{v}=\Pi \left(L_{e,k}^{v}\right)$ and transformed into the information of probability domain. Finally, it is re-mapped to the symbol message as the a priori information of the MPA detector.

(20) $$P\left(s_{v}^{l}\right)=\prod_{m=1}^{Q}\frac{{\left(L_{a}^{v,\left(l-1\right)Q+m}\right)}^{1-q_{v}^{m}}}{1+L_{a}^{v,\left(l-1\right)Q+m}},$$

where $q_{v}^{m}\in \left\{0,1\right\}$.

### 4.3. Update Variable Nodes of MPA Decoder

The variable nodes update their information when they receive the prior information $P\left(s_{v}^{l}\right)$, and then pass it to the neighboring function nodes. After this processing, the iteration between the MPA detector and coupled polar decoder is complete. The message can be updated by

(21) $$M_{q_{j}\to g_{i}}\left(s_{v}^{l}\right)=P\left(s_{v}^{l}\right)\cdot \prod_{t\in \left\{Q_{j}\backslash i\right\}}M_{g_{t}\to q_{j}}\left(s_{t}^{l}\right).$$

Although the coupled polar decoding provides better performance than SCAN algorithm, it also increases the complexity of decoding. For base polar code, the complexity of the SCL decoder is $O\left(L_{o}N_{m}\log_{2}\left(N_{m}\right)\right)$, where $N_{m}$ is the mother code length. Obviously, the complexity is proportional to the list size $L_{o}$. Thus, the basic idea of reducing complexity is to keep the list size of each user’s decoder as small as possible. We note that, in fact, when the channel conditions are good, the SCL decoder can decode correctly with a smaller list size. Therefore, a smaller list size $L_{\min}$ is given to each CB at the beginning of the iteration and is maintained in subsequent iterations if it is decoded correctly. In other words, only those CBs that fail to decode increase the list size of the decoder in the next iteration. When all users decode correctly, the iterative processing is terminated. Additionally, a maximum list size $L_{\max}$ is given to avoid excessive complexity. The list size $L_{v,k}$ of error CBs can be modified by

(22) $$L_{v,k}=\min\left(L_{\max},2L_{v,k}\right).$$

Thus, we summarize the JIDS algorithm for PIC polar-coded SCMA system as Algorithm 3.
**Algorithm 3** Joint iterative detection and SCL decoding**Input**: Signal $\left\{y_{1},\ldots ,y_{l},\ldots ,y_{L}\right\}$ from the channel, maximum number of iterations $I_{\max}$, windowed size $L_{w}$, list size $L_{\min}$ and $L_{\max}$.**Output**: The decoding decision $\hat{U}=\left\{{\hat{u}}_{1},{\hat{u}}_{2},\cdots ,{\hat{u}}_{V}\right\}$.  1: Initialize: $M_{q_{j}\to g_{i}}=1/M$ and $L_{v,k}=L_{\min}$, 1≤v≤V, $1\leq k\leq L_{c}$.  2: **for**
*iter_num* = $1\to I_{\max}$
**do**  3: **for**
l=1→L
**do **//Update the function nods  4: **for**
v=1→V
**do**  5: Calculate the extrinsic messages $L_{e,\mathrm{MPA}}^{v}$ by (4) to (6).  6: **end for**  7: **end for**  8: **for**
v=1→V
**do** //Update the Priori Information  9: De-interleave the $L_{e,\mathrm{MPA}}^{v}$ to $z_{v}$, and input to the coupled polar decoder.10: Run the decoder, calculate the extrinsic messages $L_{e}^{v}$ by HC algorithm.11: **for**
$k=1\to L_{c}$
**do**12: **if** CRC check fails **then**13: Modify the list size by (22).14: **end if**15: **end for**16: Interleave the $L_{e}^{v}$ to $L_{a}^{v}$.17: **end for**18: **for**
l=1→L
**do**19: **for**
v=1→V
**do**20: Transform the priori information $L_{a}^{v}$ into the probability domain by (20).21: Update the information of variable nodes by (21).22: **end for**23: **end for**24: If all users decode correctly, exit the iteration.25: **end for**

## 5. Optimization of Partially Information-Coupled Polar Code Based on EXIT Idea

In this section, the partially information-coupled polar code is optimized based on EXIT idea. The effects of weight factor α, impact of couple rate δ, and performance comparison of different code are analyzed based on transfer characteristics (TC) chart. Additionally, searching for the optimal value of α and optimization of couple rate δ for the PIC PCCA-polar code are performed in this section. We use the EXIT idea to guide our search for the optimal design δ-value, which is the key parameter of the PIC PCCA-polar code.

### 5.1. Transfer Characteristics of Base Polar Decoder

As shown in Figure 1, the MPA detector converts the channel information and the prior information into the extrinsic information, which is interpreted as the prior information of the coupled polar decoder. This messages passing process complies with “Turbo Principle,” which is very common in iterative system. The EXIT chart can predict the behavior of the iterative decoder by solely analyzing the individual constituent decoders. The transmission characteristic curve of channel decoder reflects the relationship between its input mutual information and output mutual information. The output mutual information of channel decoder is corresponding to the input mutual information of MPA detector. Importantly, the higher the output mutual information of channel decoder is, the higher the input mutual information of MPA detector is, and the better the performance of SCMA system. Based on the EXIT idea, in this section, we focus on the design of PIC polar code by the TC chart.

We use $I_{A}$ to denote the mutual information between a prior information *Z* and BPSK-modulated symbols *X* and use $I_{E}$ to denote the mutual information between a prior information $L_{e}$ and BPSK-modulated symbols *X*, respectively. According to [29], *Z* can be seen as a Gaussian-distributed variable with the mean $\mu_{z}=\sigma_{z}^{2}/2$ and the variance $\sigma_{z}^{2}$. The conditional probability density function can be expressed as

(23) $$p_{z}\left(\tau |X=x\right)=\frac{1}{\sqrt{2\pi }\sigma_{z}}\exp\left(\frac{-{\left(\tau -\mu_{z}x\right)}^{2}}{2\sigma_{z}^{2}}\right).$$

So the input mutual information $I_{A}$ can be calculated as follows [29]

(24) $$I_{A}\left(\sigma_{z}\right)=1-\int_{-\infty }^{+\infty }\frac{1}{\sqrt{2\pi }\sigma_{z}}\exp\left(-{\left(\tau -\mu_{z}\right)}^{2}/2\sigma_{z}^{2}\right)\cdot \log_{2}\left(1+e^{-\tau }\right)d\tau .$$

The output mutual information $I_{E}$ is denoted as

(25) $$I_{E}=\frac{1}{2}\sum_{x=\pm 1}\int_{-\infty }^{+\infty }p_{z}\left(\tau |x\right)\log_{2}\frac{2\cdot p_{z}\left(\tau |x\right)}{\sum_{x^{\prime }=\pm 1}p_{z}\left(\tau |x^{\prime }\right)}d\tau .$$

Viewing $I_{E}$ as a function of $I_{A}$, the extrinsic information transfer characteristics of polar decoder can be defined as

(26) $$I_{E}=\Omega \left(I_{A}\right).$$

**Figure 9:**
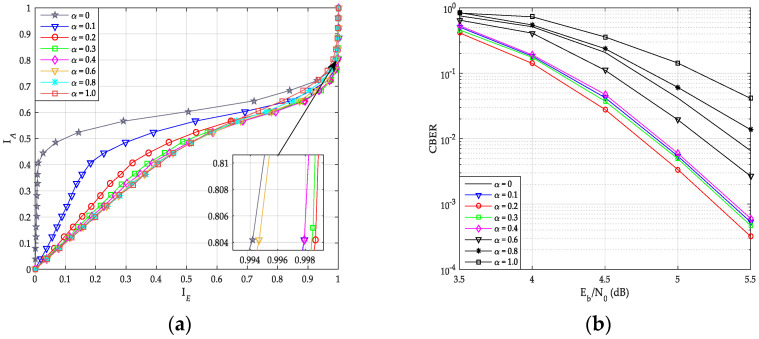
Performance of systematic polar code with SCAN decoder. (**a**) Transfer characteristics; (**b**) code block error rate (CBER) of PC-SCMA over additive white Gaussian noise (AWGN) channel.

Figure 9a,b shows the TC chart and CBER curves of uncoupled systematic PCCA-polar code with SCAN decoder over AWGN channel, respectively. Base polar code is constructed by Bhattacharyya parameter-bound method proposed in [8], weight factor α is taken from 0 to 1, and the number of out loop iterations is 5. The information bits length *K* = 100 and code rate *R* = 1/2. From Figure 9a, the TC curve is closer to the right, the better performance of decoder is ($I_{E}=1$ represents the largest output mutual information). As shown in Figure 9b, the best CBER performance is the decoder with α=0.2, and its corresponding TC curve is also the rightmost in Figure 9a. In addition, both the TC chart and the CBER curves show that the decoders with α = 0.1, 0.3, and 0.4 have very similar performance. It can be seen, the performance of CBER is consistent with the analysis of TC chart. However, compared with the growth of the code length *N*, finding the optimal α-value based on TC chart analysis is less complex.

**Figure 10:**
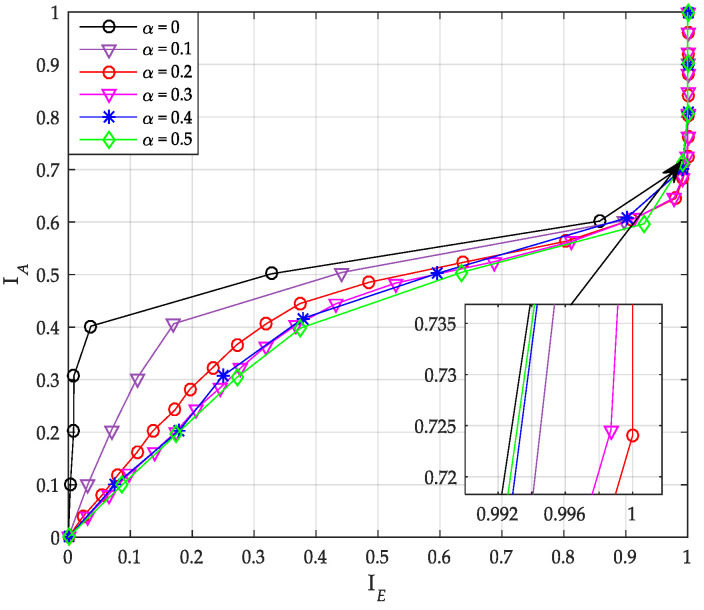
Transfer characteristics of systematic polar code with successive cancellation list (SCL) decoder.

The extrinsic message of SCL decoder is constructed by HC algorithm in Section 4. In HC algorithm, when the CRC check fails, the SCAN algorithm is used to construct the extrinsic messages. Therefore, the iterative performance of the SCL decoder is also affected by the α-value in SCMA system. The transfer characteristics of uncoupled systematic PCCA-polar code with SCL decoder is given by Figure 10. From the figure, the optimal value of α is 0.2.

### 5.2. Optimization of Coupling Ratio δ for PIC PCCA-Polar Code

In Section 3, we introduced a coupling ratio δ, but did not explain what is the optimal value of the factor. In this subsection, we use the TC chart to analyze the impact of the factor δ and guide our search for the optimal δ-value. Factor δ is taken from 0 to 1, the information bits length is set to *K* = 64, 100 and the code rate is *R* = 1/3, 1/2. 

**Figure 11:**
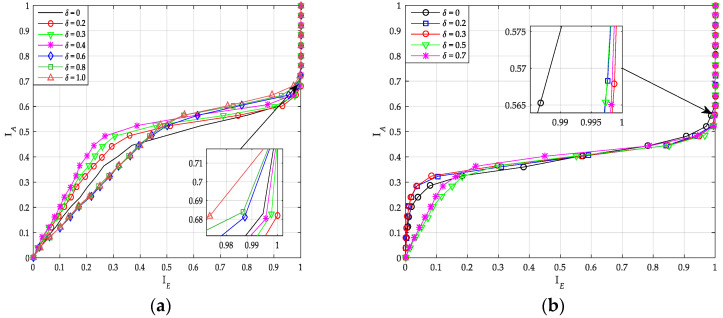
Transfer characteristics of PIC PCCA-polar code with different δ. (**a**) *K* = 100, *R* = 1/2; (**b**) *K* = 64, *R* = 1/3.

As shown in Figure 11a, the curves with coupling ratio δ = 0.2 and 0.3 are the rightmost, and have similar performance. For the coupled code, high coupling ratio corresponds to high decoding complexity (the higher base code rate). Thus, we can just choose δ = 0.2 as the optimal value for PIC polar when *K* = 100 and *R* = 1/2. Similarly, when *K* = 64 and *R* = 1/3, we choose δ = 0.3 as the optimal value for PIC polar based on Figure 11b.

**Figure 12:**
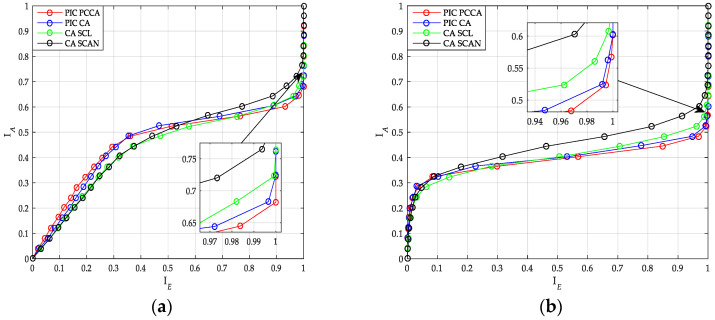
Transfer characteristics of polar code. (**a**) *K* = 100, *R* = 1/2; (**b**) *K* = 64, *R* = 1/3.

Based on the previous optimization results, Figure 12 shows the transfer characteristics of PIC polar codes and uncoupled polar codes. The performance of uncoupled polar code with SCAN decoder is worst among other codes. At the same time, the performance of PIC PCCA-polar code is the best. Additionally, when the input mutual information is small, code blocks that are coupled to each other cannot exchange correct extrinsic messages, the output mutual information of PIC polar code (whether it’s PCCA-based or CA-based) is smaller than that of the uncoupled polar code.

## 6. Performance Evaluation

In this section, the simulation results are provided to evaluate the block (TB and CB) error rate performance and complexity of the uplink SCMA system over the AWGN channel and Rayleigh fading channel. Assuming there are six users multiplexed over four orthogonal resources, the overloading is 150%. The mapping matrix has already been given in (1), and the corresponding SCMA codebook is designed according to [30]. Base systematic polar code is constructed by Bhattacharyya parameter-bound method. For each user, the base code length is set to *N* = 192, 200, and the code rate is *R* = 1/3, 1/2. To ensure that systematic PCCA-polar code and CA-polar code have the same number of assistant bits, the CRC-8 with the generator polynomial $g\left(x\right)=x^{8}+x^{2}+x+1$ and CRC-11 with the generator polynomial $g\left(x\right)=x^{11}+x^{10}+x^{9}+x^{5}+1$ are used in PCCA-polar code and CA-polar code, respectively. The number of parity check bits $n_{pc}$ is set to 3. The weight factor α and coupling ratio δ are selected according to the values optimized in Section 5. The number of blocks in a TB and the window size of coupled polar decoder are set to $L_{c}=10$ and $L_{w}=10$. For the base SCL decoder, the minimum and maximum list sizes are configured as $L_{\min}=4$ and $L_{\max}=32$. Additionally, the rate-matching pattern is obtained according to the introduced method of [14]. As the comparative scheme, the LDPC encoder and rate-matching algorithm used in 3GPP TS 38.212 are employed, where the CRC bits are generated by CRC-11. In addition, the log-Belief Propagation (log-BP) algorithm with 30 inner-loop iterations is used for LDPC decoding. In the receiver, the MPA detection within one inner-loop iteration is employed for MUD, and the number of outer-loop iteration between the MPA detector and channel decoder is set to 5.

### 6.1. Block Error Rate Performance Comparison 

**Figure 13:**
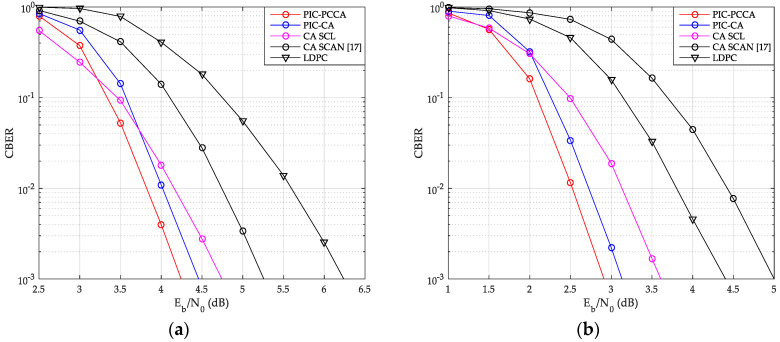
CBER of SCMA over AWGN channel. (**a**) *K* = 100, *R* = 1/2; (**b**) *K* = 64, *R* = 1/3.

**Figure 14:**
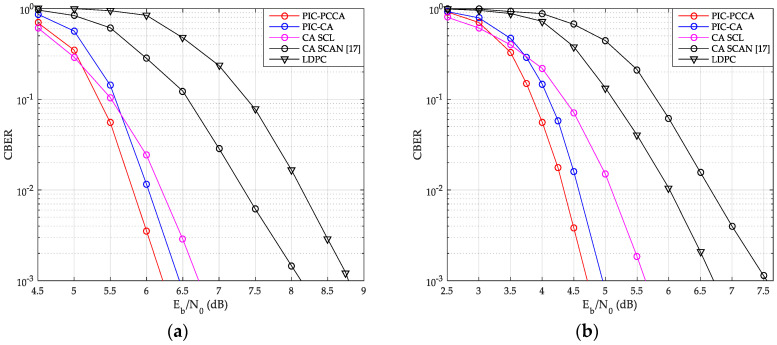
CBER of SCMA over Rayleigh channel. (**a**) *K* = 100, *R* = 1/2; (**b**) *K* = 64, *R* = 1/3.

As shown in Figure 13 and Figure 14, we have compared the CBER performance of SCMA system with PIC polar codes, uncoupled polar codes, and LDPC code over AWGN and Rayleigh channels.

From these figures, the PIC PCCA-PC-SCMA has the best performance at various code rate and channel configurations, e.g., over AWGN channel, when K = 100 and R = 1/2, the PIC CA-PC-SCMA achieves 0.28 dB coupling gain over the CA (SCL)-PC-SCMA at CBER = 10^−3^ as shown in Figure 13a. In the early stage of each CB decoding, parity-check bits are used to correct errors and save the correct decoding path as far as possible. Therefore, PIC PCCA-polar code also achieved an additional 0.22 dB coding gain compared to PIC CA-polar code over SCMA system. Similarly, for the code rate-1/3, PIC PCCA-PC-SCMA can also obtain corresponding gain as shown in Figure 13b. However, at the same time, the performance of uncoupled CA-PC-SCMA based on SCAN decoding is the worst among other systems. Since error blocks cannot provide perfect extrinsic messages to adjacent blocks in a TB, adjacent CB can only be decoded at a higher code rate. In particular, in the lower $E_{b}/N_{0}$ range, a wrong CB often causes continuous CB errors in a same TB, in other words, these errors are aggregated. Thus, with SCL decoding (for CB), the CBER performance of PIC PC-SCMA systems is worse than that of uncoupled PC-SCMA system. Similarly, we can obtain the same results from Figure 14.

**Figure 15:**
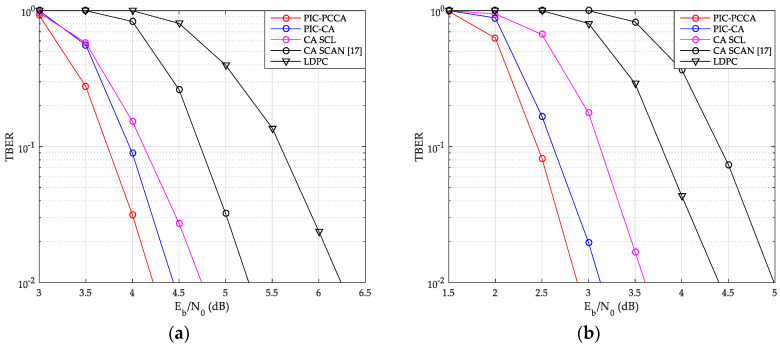
TB error rate (TBER) of SCMA over AWGN channel. (**a**) *K* = 100, *R* = 1/2; (**b**) *K* = 64, *R* = 1/3.

**Figure 16:**
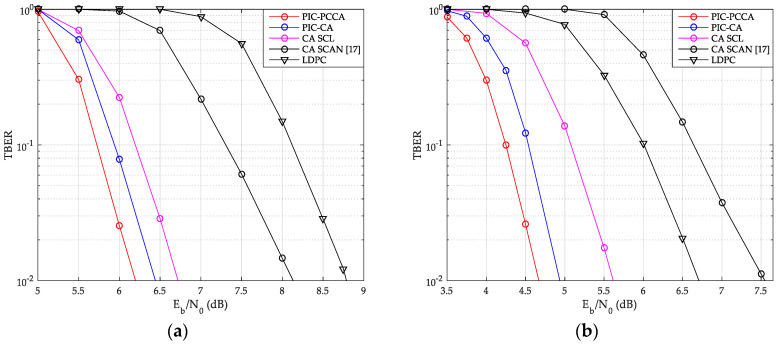
TBER of SCMA over Rayleigh channel. (**a**) *K* = 100, *R* = 1/2; (**b**) *K* = 64, *R* = 1/3.

The TBER performance curves of SCMA system are also given by Figure 15 and Figure 16. Obviously, we can get results similar to CBER performance. At a low $E_{b}/N_{0}$, although wrong CB may easily cause continuous CB errors in a same TB, the look-back and go-back scheme can also be used to improve the probability of correct decoding once any CB is correctly decoding. PCCA-polar code uses parity check to improve the performance of the base code, thus achieving the goal of reducing TBER. Therefore, even at a low $E_{b}/N_{0}$, PIC PCCA-PC-SCMA also maintains the best TBER performance. Table 2 shows the specific performance gains of PIC PCCA-PC-SCMA for various code rates and channel configurations at target CBER = 10^−3^ and TBER = 10^−2^.

### 6.2. Complexity Analysis 

For a PC-SCMA system, the complexity (CB-based) can be given by $\bar{I}\left(O\left(Jd_{f}M^{d_{f}}\right)+\bar{L}O\left(N_{m}\log_{2}\left(N_{m}\right)\right)\right)$, where $\bar{I}$ is the average number of iterations of JIDS, $d_{f}$ is the degree of the SCMA function nodes and $\bar{L}$ is the average list size per iteration of CB (including feed-back decoding of PIC polar code). Additionally, the list size of the SCAN algorithm is treated as 1. Thus, the complexity of PC-SCMA system can be represented by the average number of iterations $\bar{I}$ and the average list size $\bar{L}$, e.g., the complexity of all kinds of PC-SCMA systems for *K* = 100 and *R* = 1/2 over AWGN channel is summarized in Table 3. 

On the basis of JIDS algorithm, at a low $E_{b}/N_{0}$, since the high block error rate, a large number of CBs are decoded with large list size. From Table 2, compared with the uncoupled PC-SCMA with SCL decoder, PIC PCCA (or CA)-PC-SCMA has the higher complexity. Especially, the PIC CA-PC-SCMA system has the highest complexity among other PC-SCMA systems. At a high $E_{b}/N_{0}$, the probability that most CBs can be decoded with a smaller list size in the feed-forward decoding process increases. Therefore, the complexity of the PIC PCCA (or CA)-PC-SCMA system decreases gradually. In particular, with the help of parity check for the base code, PIC PCCA-SCMA can be decoded with fewer outer-loop iterations. Thus, with SCL decoder, the complexity of PIC PCCA-PC-SCMA is lower than that of uncoupled PC-SCMA. By the way, although the PC-SCMA presented in [17] has the lowest complexity, its performance is far from that of other PC-SCMA schemes.

## 7. Conclusions

In this paper, the uplink PC-SCMA system is improved by information coupling and parity check. The systematic PCCA-polar code is encoded as the base code of the PIC polar code. A low-complexity joint iterative detection and SCL decoding receiver is proposed for the SCMA system. The hybrid construction algorithm is designed to construct the extrinsic messages of coupled polar decoder. Based on EXIT idea, we have optimized the weight factor of extrinsic messages construction algorithm and coupling ratio of PIC PCCA-polar code. The simulation results show that, at various code rate and channel configurations, the PIC PCCA-PC-SCMA has the best performance in TBER and CBER. In addition, compared with uncoupled PC-SCMA with SCL decoder, the complexity of PIC PCCA-PC-SCMA is reduced at a high $E_{b}/N_{0}$. 

## Figures and Tables

**Table 1:** Summary of abbreviations.

Abbreviation	Full Name	Abbreviation	Full Name
5G	Fifth generation	AWGN	Additive white Gaussian noise
BC	Bayes construction	BLER	Block error rate
BMC	Binary-input memoryless channel	BP	Belief propagation
CA	CRC-aided	CB	Code block
CBER	Code block error rate	CRC	cyclic redundancy check
DCA	Distributed CRC-aided	eMBB	enhanced Mobile BroadBand
EXIT	Extrinsic information transfer	FN	Function node
HC	Hybrid construction	IDD	Iterative detection and decoding
JIDD	Joint iterative detection and decoding	JIDS	Joint iterative detection and SCL decoding
JSC	Joint successive cancellation	LDPC	Low-density parity-check
LDPC-SCMA	LDPC coded sparse code multiple access	LDS	Low-density signatures
LLR	Log-likelihood rate ratio	log-BP	log-Belief propagation
mMTC	massive Machine Type Communications	MPA	Message passing algorithm
MUD	Multi-user detection	MUSA	Multi-user shared access
NOMA	Non-orthogonal multiple access	NR	New radio
OMA	Orthogonal multiple access	PCCA	Joint parity check and CRC aided
PC-SCMA	Polar-coded sparse code multiple access	PDMA	Pattern division multiple access
PE	Processing element	PIC	Partially information-coupled
QoS	Quality of service	SC	Successive cancellation
SCAN	Soft cancellation	SCL	Successive cancellation list
SCMA	Sparse code multiple access	SISO	Soft-input-soft-output
TB	Transport block	TBER	TB error rate
TC	Transfer characteristic	URLLC	Ultra-Reliable and Low Latency Communications
VN	Variable node		

**Table 2:** Gain of PIC PCCA-PC-SCMA vs. other SCMA systems (dB).

Parameters			Polar			LDPC
			PIC CA	CA (SCL)	CA (SCAN) [17]	
AWGN	*K* = 100 *R* = 1/2	CBER	0.22	0.5	1	2
		TBER	0.22	0.52	1.03	2.02
	*K* = 64 *R* = 1/3	CBER	0.22	0.7	2.09	1.49
		TBER	0.25	0.75	2.12	1.52
Rayleigh	*K* = 100 *R* = 1/2	CBER	0.23	0.5	1.91	2.57
		TBER	0.25	0.52	1.93	2.59
	*K* = 64 *R* = 1/3	CBER	0.24	0.92	2.84	2
		TBER	0.27	0.95	2.88	2.04

**Table 3:** The average number of iterations $\bar{I}$ and the average list size $\bar{L}$ over AWGN channel.

$E_{b}/N_{0}$ (dB)		PIC PCCA	PIC CA	CA (SCL)	CA (SCAN) [17]
2.5	$\bar{I}$	5	5	5	5
	$\bar{L}$	17.83	18.27	17.78	1
3	$\bar{I}$	5	5	5	5
	$\bar{L}$	16.2	17.89	14.94	1
3.5	$\bar{I}$	4.79	5	4.96	5
	$\bar{L}$	11.74	14.42	11.35	1
4	$\bar{I}$	3.85	4.39	4.42	5
	$\bar{L}$	8.08	10.1	8.45	1
4.5	$\bar{I}$	2.91	3.34	3.55	4.95
	$\bar{L}$	6.24	7.17	6.8	1

## Appendix A

**Proof** **of** **Theorem** **1.** Denote $N_{m}=2^{n}$ as the mother code length, $G\left(n\right)={\left[\begin{array}{cc} 1 & 0\\ 1 & 1 \end{array}\right]}^{⊗n}$ as the generator matrix, $g_{n,i}$ as the *i*-th row of G(n), and $1_{n}^{i}$ as the indices of ones in $g_{n,i}$. Considering the case of $A^{\prime }=\left\{1,\ldots ,N_{m}\right\}$, when n=1, there is $G\left(1\right)=\left[\begin{array}{cc} 1 & 0\\ 1 & 1 \end{array}\right]$. Obviously, if $j\in 1_{1}^{i}$, there is $1_{1}^{j}\subseteq 1_{1}^{i}$. Suppose that the relation holds when n=r>1 that is, if $j\in 1_{r}^{i}$, then $1_{r}^{j}\subseteq 1_{r}^{i}$. When n=r+1, the generator matrix can be represented by

(A1) $$G\left(r+1\right)=\left[\begin{array}{cc} G\left(r\right) & 0\\ G\left(r\right) & G\left(r\right) \end{array}\right].$$

From (A1), if $1\leq i\leq 2^{r}$, there is $1_{r+1}^{i}=1_{r}^{i}$. So, if $j\in 1_{r+1}^{i}$, the relation $1_{r+1}^{j}\subseteq 1_{r+1}^{i}$ holds. If $2^{r}+1\leq i\leq 2^{r+1}$, the $1_{r+1}^{i}$ can be expressed as $1_{r+1}^{i}=1_{r}^{i-2^{r}}\cup \left\{j+2^{r}|j\in 1_{r}^{i-2^{r}}\right\}$. If $j\in 1_{r+1}^{i-2^{r}}$, we can obtain the relations $1_{r+1}^{j}=1_{r}^{j}\subseteq 1_{r}^{i-2^{r}}\subset 1_{r+1}^{i}$ and $1_{r+1}^{j+2^{r}}=1_{r+1}^{j}\cup \left\{j^{\prime }+2^{r}|j^{\prime }\in 1_{r}^{i-2^{r}}\right\}\subseteq 1_{r+1}^{i}$. Thus, the relation $1_{r+1}^{j}\subseteq 1_{r+1}^{i}$ holds if $j\in 1_{r+1}^{i}$. Here, we have proved that theorem 1 holds for generating matrix G(n). The indices of information with assistant bits (including CRC bits and parity check bits) is represent by $A^{\prime }$, and the indices of frozen bits is ${A^{\prime }}_{c}$. Denote ${A^{\prime }}_{i}$ as the *i*-th element of $A^{\prime }$, $g_{i}$ as the *i*-th row of $G_{A^{\prime }A^{\prime }}$, and $1^{i}$ as the indices of ones in $g_{i}$. We can get the result $1^{i}=\left\{1_{n}^{{A^{\prime }}_{i}}\backslash k|k\in {A^{\prime }}_{c}\right\}$. According to the previous analysis of the generation matrix G(n), the relation $1_{n}^{{A^{\prime }}_{j}}\subseteq 1_{n}^{{A^{\prime }}_{i}}$ can be obtained. Thus, if $j\in 1^{i}$, the relation $1^{j}\subseteq 1^{i}$ holds.  □
